# Galvanic corrosion protection of Al-alloy in contact with carbon fibre reinforced polymer through plasma electrolytic oxidation treatment

**DOI:** 10.1038/s41598-022-08727-7

**Published:** 2022-03-16

**Authors:** Junyi Liu, Xiaohu Huang, Yi Ren, Lai Mun Wong, Hongfei Liu, Shijie Wang

**Affiliations:** Institute of Materials Research and Engineering (IMRE), A*STAR (Agency for Science, Technology and Research), 2 Fusionopolis Way, Singapore, 138634 Singapore

**Keywords:** Chemistry, Engineering, Materials science

## Abstract

Al-alloy/carbon fibre reinforced polymer (CFRP) joint systems offer exceptionally lightweight, superior fatigue behaviour and impact resistance for aerospace applications. Nevertheless, the galvanic corrosion at the joint interfaces accelerates the adhesive failure and strength damage. In this work, oxidation of Al 7075 alloy was studied by employing plasma electrolytic oxidation (PEO) and thin film sulphuric acid anodizing (TFSAA) methods, addressing their galvanic corrosion (GC) protection performance in contact with CFRP. Structural and electrochemical characterisations were carried out in tandem with varied oxidation process parameters, revealing that high voltage PEO resulted in crystallized compact ceramic coating and thus improved GC protection. A decrease in the GC current by ~ 90% has been achieved by using the PEO coating at 700 V compared with the ~ 12% current reduction of commercial TFSAA coating. Further microstructure studies revealed that the improved GC protection of the crystallized PEO coating was realized by suppressing the initiation and propagation of localized pitting due to the improved electrical isolation between the Al-alloy/CFRP interfaces. A high voltage PEO process provides sufficient energy to produce uniform and crystalline ceramic coating consisting of Al_2_O_3_ and mullite, which give rise to improved corrosion protection.

## Introduction

Fibre-reinforced polymer (FRP) composites are widely used in various engineering areas due to their unique properties, typically the lightweight, high specific stiffness, improved tailorability, and high corrosion resistance. Their incorporation into structural materials, forming adhesion bonding and/or mechanical joining to the host structures, can significantly reduce the weight while improve the mechanical performance. Such reinforced structures widely broadened the fit for purpose structural designs, particularly in aerospace and marine applications^1–4^.

Aluminium (Al)-alloy/carbon fibre reinforced polymer (CFRP) joint systems have been widely applied in aerospace industry, e.g., to fabricate the inner/outer flaps and the outer wings of airplanes^1,5,6^. As a promising material candidate for structural applications, the Al-alloy/CFRP systems combine the high strength of metallic materials and the lightweight of CFRPs, offering enhanced fatigue life and increased impact resistance. However, the implementation of Al-alloy/CFRP system brings up new challenges: the galvanic corrosion (GC) of Al alloy in contact with CFRP. In recent years, the GC behaviours of Al alloys have been extensively studied in contact with various metals, such as Al/Fe, Al/stainless steel, Al/Cu, Al/Mg, etc.^7–10^. The GC of Al-alloy in contact with CFRP has also been studied^2,4,11^. For example, Zou et al. investigated the corrosion effect of Al alloy in contact with CFRP in a 3.5 wt% NaCl solution and observed a severe selective corrosion on the surface of Al alloy 2219 and ZL205A^11^. Such selective corrosion may cause the damage of the Al-alloy/CFRP joining structures and thus degrade the mechanical properties of the components. In this regard, it is necessary to improve the corrosion resistance by suitable surface treatment of Al alloy, to electrically separate the Al-CFRP contact.

Chemical conversion, anodization, electrochemical deposition, and sol–gel process are currently the most popular surface treatment methods that have been used for Al alloys to reduce their corrosion susceptibility^12–16^. Among them, anodization produces a thin oxide film on the surface of base material with excellent adhesion and high resistance to hydration. These characters of Al anodization make it extensively used in the aerospace industry in the past few decades^17^. Plasma electrolytic oxidation (PEO) is evolved from the traditional anodization process, but with a much higher working voltage^18–20^. PEO coatings are quite promising for light alloys (Al- and Mg- and Ti-based ones) treatments, not only to improve their corrosion protections but also to enhance their wear resistance^21–31^. Through PEO process, a ceramic coating can be intrinsically formed on the surface under the instantaneous high temperature and strong electric field generated by sparking discharge^32,33^. Although the effect of PEO coatings on the general corrosion and tribological behavior of light alloys is well documented in the literature, the GC protection effect of such coating on the couples of Al-alloy/CFRP is still lacking, neither has PEO coating properties been optimized for the Al-alloy/CFRP applications.

In this work, with the zero-resistance ammeter (ZRA) method, we studied the GC behaviour between CFRP and Al 7075, which is a widely used metal-fibre system in aerospace industry. Structural and electrochemical characterizations were carried out to compare the effects of PEO coating and traditional anodization coating on the GC behaviour of the Al 7075 alloy in contact with CFRP. The effect of the PEO process voltage on the structural and compositional properties and therefore the GC protection performance was further studied, providing an optimization for the protection of Al 7075 in the Al/CFRP coupling.

## Results and discussion

### Electrochemical and corrosion property of Al-alloy/CFRP coupling

**Figure 1:**
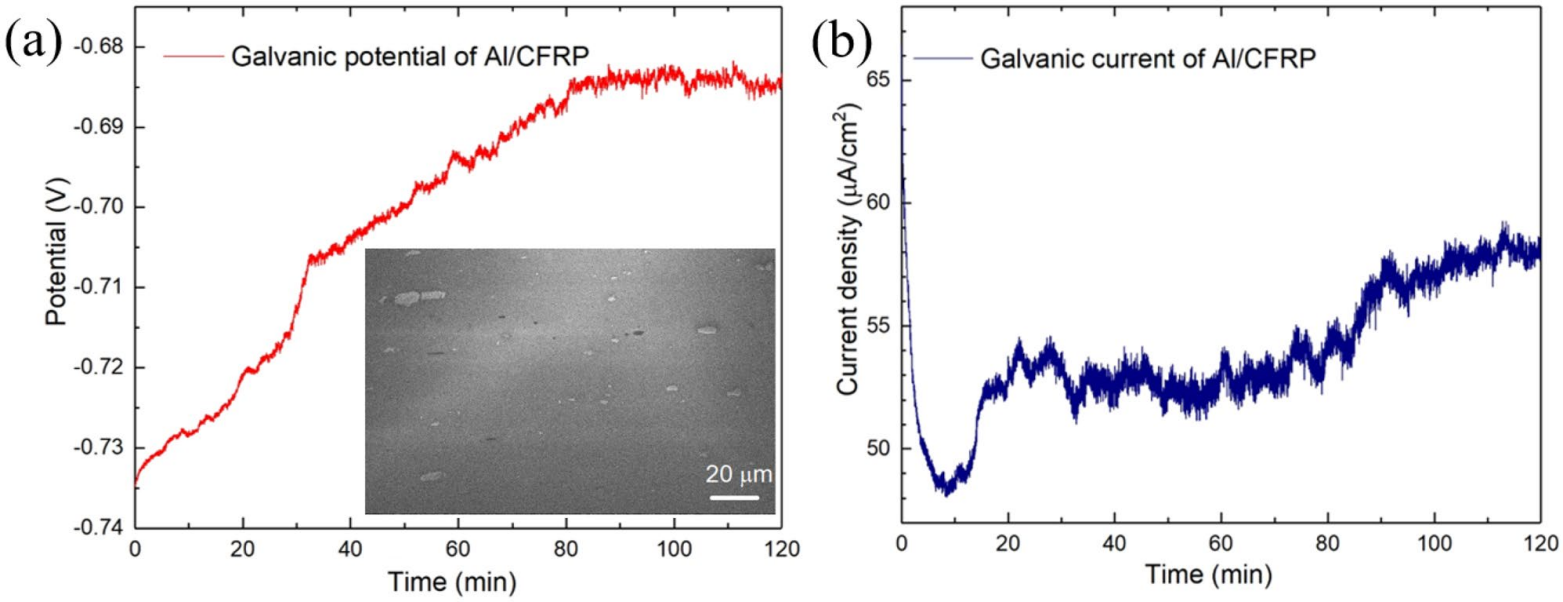
Time-dependent variations in the (**a**) galvanic electrode potential with the insert SEM image showing the intermetallic precipitates and (**b**) galvanic current density of the Al/CFRP connected in 3.5 wt% NaCl.

Al 7075 alloy has been extensively used in aerospace industry due to its high strength provided by the added strengthening phases^34^. They form several intermetallic precipitates with the size ranging from 1.0 to 20.0 μm as shown in the insert of Fig. 1a, which play important roles in the mechanical properties of the alloy. EDX mapping of the Al 7075 alloy before the corrosion test indicates that most of the intermetallic precipitates consisted of Al, Cu, Fe and Mg with varied compositions (Fig. S1 of Supplementary Material). The coarse particles are most likely Al_7_Cu_2_Fe and (Al,Cu)_6_(Fe,Cu), which are commonly formed during the solidification of Al 7075 alloy, while Mg_2_Si and MgZn_2_ intermetallics are of much smaller quantities^35–38^.

Figure 1a and b shows the variations in the galvanic electrode potential and current density of Al-alloy/CFRP coupling as a function of time. CFRP has more positive standard electrode potential (0.52 V) when compared with most metals (negative standard electrode potential). When CFRP was coupled to Al 7075, it served as the cathode with oxygen reduction and/or hydrogen evolution through the cathodic reactions. Likewise, Al 7075 served as the anode, where accelerated dissolution takes place driven by galvanic reactions^4^. The Al-alloy/CFRP couplings started with a high current density (65.7 μA/cm^2^) as shown in Fig. 1b. The current density rapidly decreased to lower than 49.0 μA/cm^2^ within 10 min, which was followed by oscillations at about 53.5 μA/cm^2^ in 20–80 min and about 58.0 μA/cm^2^ in 90–120 min, respectively. The current oscillation was typically due to the initiation of the localized corrosions and their re-passivation on the surface of the Al alloy^8^. In the initial stage of the corrosion, the high-density corrosion products tended to improve the performance of the electronic shielding as compared with that of the bare Al alloy. As the corrosion proceeded, the galvanic potential of Al-alloy/CFRP approached a steady state (i.e., − 0.685 V) at around 100 min and the corresponding galvanic current was around 58.2 μA/cm^2^. The corrosion reactions stabilized gradually with reduced fluctuations in the instantaneous current density. This indicates that the corrosion products on the surface can serve as corrosion passivator for the underneath Al alloy and they increased with the corrosion time.

**Figure 2:**
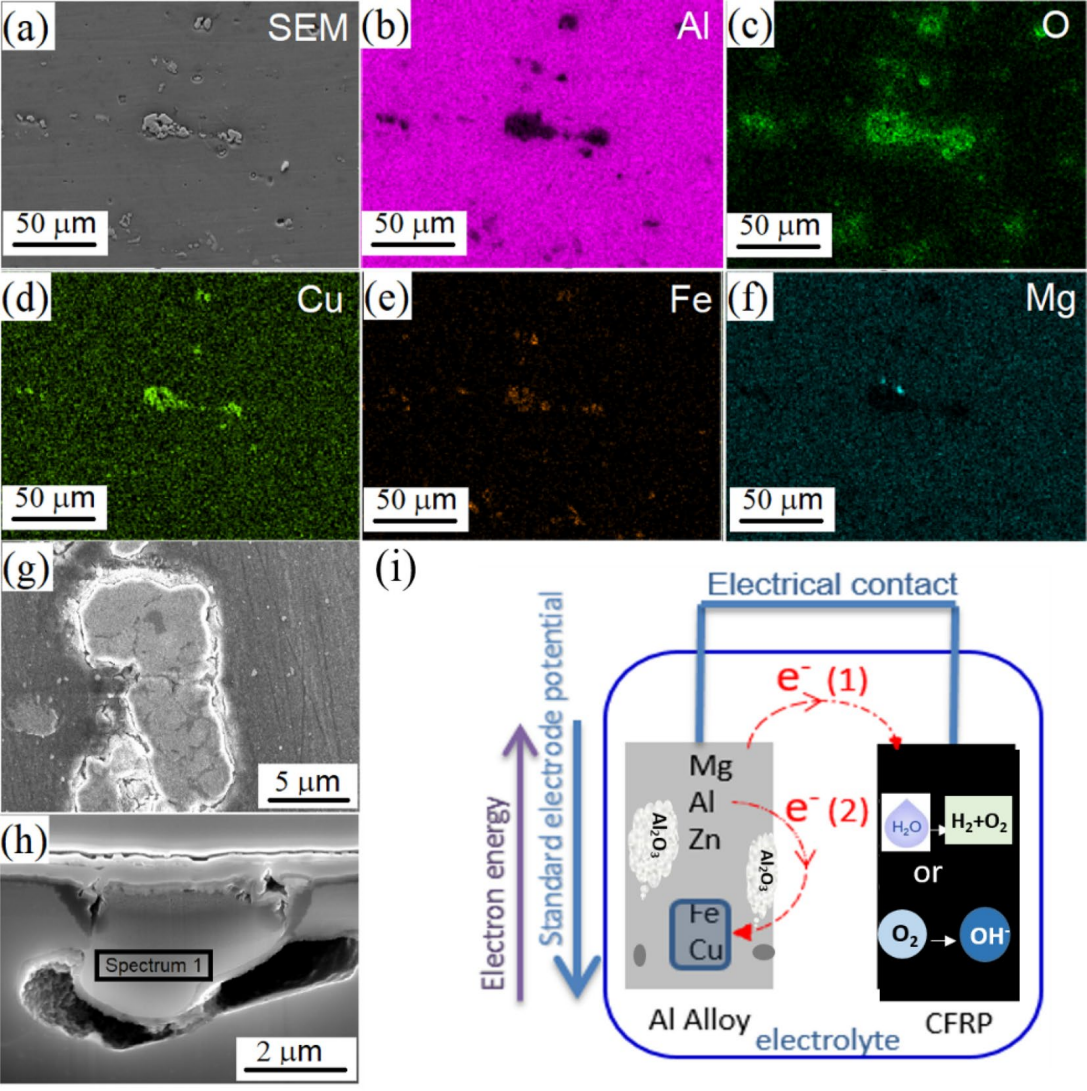
SEM microstructures (**a**) and EDX element mapping (**b**–**f**) of the Al 7075 alloy after galvanic coupling with CFRP in 3.5 wt% NaCl solution for two hours; Top view (**g**) and (**h**) cross sectional SEM images of the localized corrosions on the Cu-rich area; (**i**) Schematic diagram showing the corrosion reactions between the Al matrix and intermetallics.

Microstructures and the elemental distributions of Al, O, Cu, Fe, and Mg in the Al alloy were measured using SEM and EDX mapping after the GC testing for two hours in the 3.5 wt% NaCl electrolyte, and they are presented in Fig. 2a–f, respectively. The results indicate that the intermetallics were the initiation sites of the localized corrosions. Figure 2g and h shows the top and cross sectional view of a typical localized corroded spot. The cross sectional EDX results (Fig. S2 of Supplementary Material) of the corroded area in Fig. 2h indicates the spot was a Cu-rich intermetallic area. Their combinations reveal that Al-oxide/hydroxide precipitated at the rim of the spot and covered the opening underneath, and there was a dissolution of the Al matrix and its resultant gap between the Al matrix and the Cu-rich intermetallics. Due to the standard electrode potential difference between the intermetallics and the Al alloy matrix, the localized corrosions occur with the dissolution of either the matrix or the intermetallics^38,39^. The localized corrosion reactions were illustrated in Fig. 2i. The intermetallics containing Cu and Fe are cathodic with respect to the matrix and hence promote the dissolution of the matrix. In comparison, the Mg-rich intermetallics are anodic with respect to the matrix and thus preferentially dissolve themselves^40^. In the current study, it is reasonable to conclude that the localized electron transfers from Al to Cu promote the dissolution of the Al matrix surrounding the Cu-rich spot.

Although the oxidation products may inhibit the exchange of ions and slow down the corrosion reactions, excessive trenching will cause mechanical detachment of the particles and their dissolution into the electrolyte. The trenching effect has been discussed extensively in the literature, however, the precise mechanism is far beyond a simple galvanic coupling that caused the anodic dissolution at the interface between cathodic intermetallics and the Al alloy matrix. The locally increased pH value as a result of oxygen reduction reactions could also attack the passive films formed on the Al alloy matrix^41,42^.

### Microstructures and phase transformations upon varied PEO process voltages

**Figure 3:**
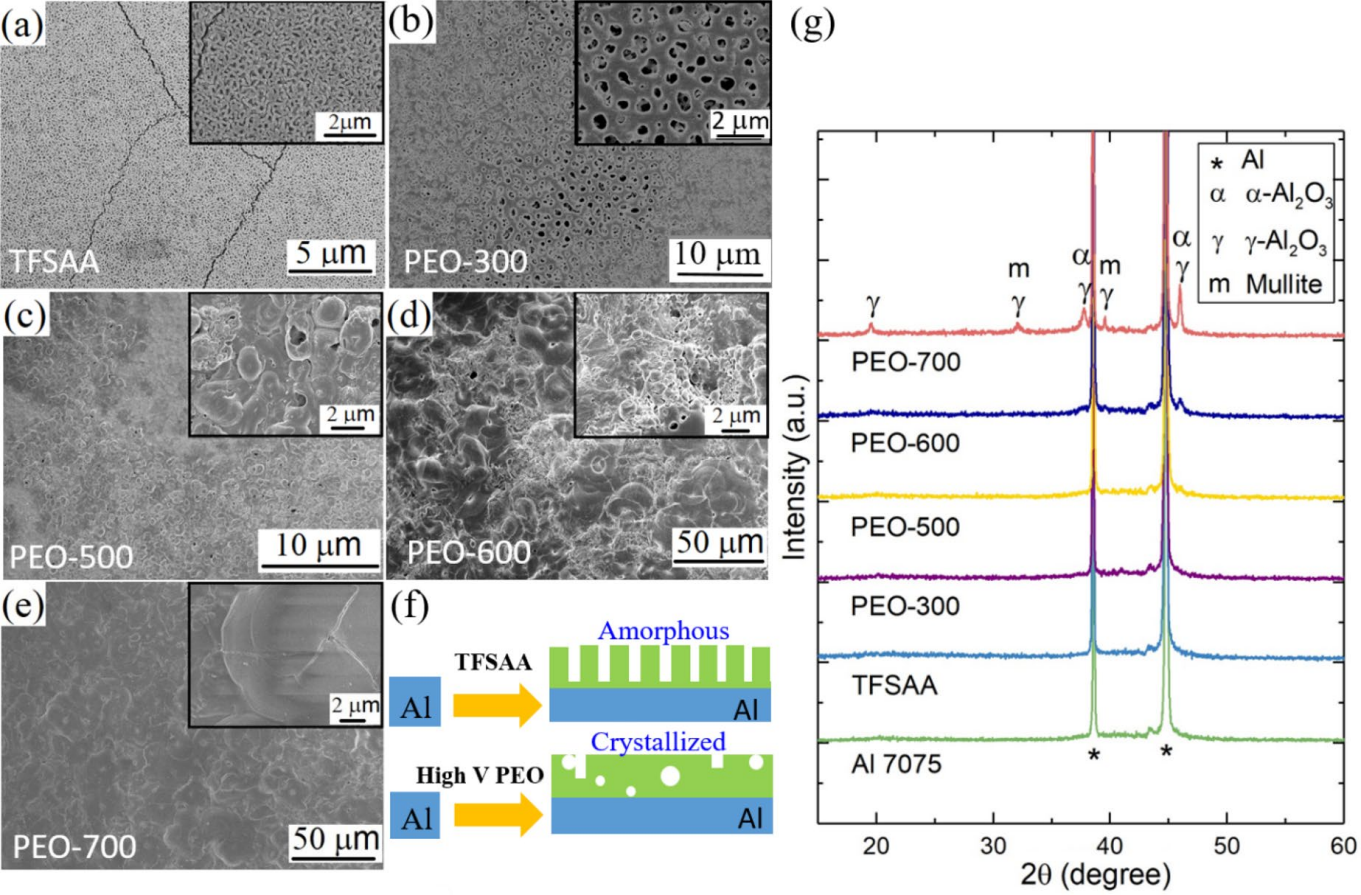
SEM images of anodized Al by (**a**) TFSAA and PEO at V_PEO_ of (**b**) 300 V, (**c**) 500 V, (**d**) 600 V, and (**e**) 700 V, respectively; (**f**) Schematic diagram showing the structure difference of TFSAA and PEO coatings (V_PEO_ = 600/700); (**g**) XRD data of the Al 7075, TFSAA and PEO coatings. The PEO coatings processed at V_PEO_ = 300, 500, 600, and 700 V were labelled as PEO-300. PEO-500, PEO-600, and PEO-700, respectively.

To study the effect of the PEO voltage as well as the comparison between the PEO and the conventional thin film sulphuric acid anodizing (TFSAA) process, 20-min oxidation treatments were carried out for both the TFSAA and PEO processes. Different phases can be formed on Al through PEO process as the electrolyte compounds vary^43^. In this work, Na_2_SiO_3_ and KOH electrolyte were employed. Some studies have shown that the SiO_3_^2−^ can enter the discharge channels leading to the increase of coating thickness and hardness, while KOH leads to the reduction in breakdown voltage and offers improvement in coating properties^44,45^. Figure 3 presents the SEM images and the XRD curves of anodized Al by TFSAA and PEO at V_PEO_ = 300–700 V, respectively. The SEM images recorded from the surface of the Al alloy processed by TFSAA and PEO at 300 V (Fig. 3a,b), show similar porous microstructures with the pore size of around 0.2 µm and 1.0 µm, respectively. The cracks in the TFSAA coating suggested tensile stresses build-up due to the different thermal expansions between Al substrate and Al_2_O_3_^46^ coating. There was no plasma generated at V_PEO_ = 300 V by visual observation. As the V_PEO_ was increased to 500 V, although onset of plasma occurred and round bumps were observed at some areas of the sample surface, the coating surface was not uniform (Fig. 3c). When the V_PEO_ was further increased, continuously plasma spreaded on the surface of the Al alloy throughout the entire process and the plasma intensity became more violent. As a result, more compact and uniform structures were formed when the V_PEO_ was increased to 600 and 700 V as those shown in Fig. 3d and e, respectively.

The XRD data were presented in Fig. 3g. Besides the XRD peaks from the Al substrate, no XRD peaks can be observed from the TFSAA coating and coatings grown at 300 V, indicating the amorphous nature of the coatings. Although plasma can be observed during the PEO-500 coating growth, hardly no XRD peaks can be distinguished from the Al background for PEO-500 coating, suggesting the coating is also amorphous or/and non-stoichiometric. The coating was partially crystallized at V_PEO_ = 600 V^47^. More crystallized structures formed at V_PEO_ = 700 V. Mullite (3Al_2_O_3_·2SiO_2_ or 2Al_2_O_3_·SiO_2_, JCPDS 15-0776) and Al_2_O_3_ (JCPDS 10-0425 and JCPDS 71-1123) were the two main constituents in the crystallized PEO coating. The mullite phase has a more compact structure than the Al_2_O_3_ in the PEO-produced coating^48^. During the PEO process, the molten alumina can react with silicate ions present in the electrolyte bath to form a stable alumina-silicate phase, i.e., the mullite^49,50^. Or they may transform to a mixture of γ- Al_2_O_3_ and α- Al_2_O_3_ according to literature^51^. The absence of the XRD peaks related to mullite or Al_2_O_3_ in the coatings produced at V_PEO_ < 500 V indicates that the magnitude of the applied voltages was not sufficient to support these phase transformations^52–54^. The structure difference of TFSAA and PEO coatings is illustrated in Fig. 3f. The TFSAA coating was amorphous and highly porous. While the ceramic PEO coatings (V_PEO_ > 600 V) showed much more compact crystallized structure with few isolated micro-pores, thus improved corrosion protection effects were expected.

### GC protections of Al alloy by the PEO coating

**Figure 4:**
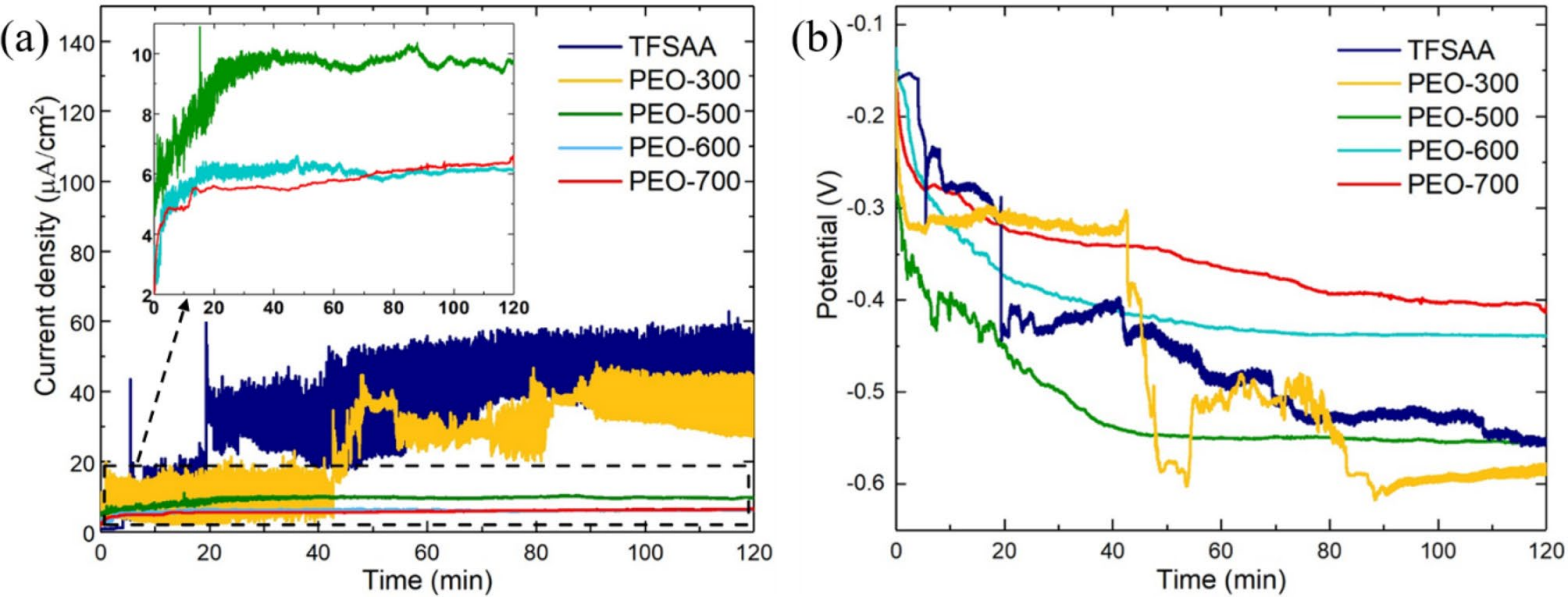
Time dependent (**a**) galvanic current densities and (**b**) electrode potentials of coated Al alloy coupled to CFRP in 3.5 wt% NaCl, the samples were processed by TFSAA at 15 V and by PEO at V_PEO_ = 300, 500, 600, 700 V.

Figures 4a shows the time-dependent galvanic current densities of the Al alloys, which were coated by TFSAA and PEO with varied V_PEO_, coupled to CFRP in 3.5 wt% NaCl. The corresponding data derived from the GC testing were summarized in Table 1. For the TFSAA and PEO-300 samples, large oscillations occurred in the current density at the initial stage and the current oscillated all the way along the testing up to 120 min. Regarding sample PEO-300 showing a more notable fluctuation, this may be caused by the larger pore size of the PEO-300 coating compared to other coatings. Larger pore size leads to wider channels for electrolyte penetration and re-passivation, thus more significant change in the potential when such events occur. The current oscillations of the anodized alloys were much more serious than that of the bare Al alloy (Fig. 1b). The highly porous structure of the anodized surface may facilitate more complex initiation and re-passivation processes by trapping the oxidized products within the pores and channels, giving rise to the increased current oscillations.

**Table 1:** The corresponding data derived from the GC testing.

Samples	Current density at 120 min (μA/cm^2^)	Electrode potential at 120 min (V)	Current density reduced up to 120 min (%)
Al 7075 T6	58.2	− 0.69	–
TFSAA	51.5	− 0.56	12
PEO-300	36.6	− 0.58	59
PEO-500	9.7	− 0.55	83
PEO-600	6.1	− 0.42	90
PEO-700	6.2	− 0.36	90

The oscillation period of galvanic current decreased with the increase of V_PEO_. On the other hand, for PEO-500 and PEO-600 samples, the current oscillations significantly reduced after GC for 60 min, indicating the stabilization of the corrosion and re-passivation; the stabilized current densities were 9.7 and 6.1 μA/cm^2^ up to 120 min, respectively. In the case of PEO-700, the current density did not exhibit apparent oscillations except the electrical noise. In this case, the galvanic current represented the anodic current of Al alloy and the current density reached to a stabilized value of 6.2 μA/cm^2^. Up to GC for 120 min, the galvanic current density of PEO-600 and PEO-700 were similar, and they were ~ 90% decreased from that of the Al-alloy/CFRP coupling without employing any anodization process. When compared with the TFSAA coating, the PEO-600 and PEO-700 samples can averagely reduce the galvanic current density by ~ 88% over the 120 min testing. The absence of galvanic current oscillations in the GC testing of the PEO-700 sample indicates an effective suppression of the pitting corrosions, which provides a promising corrosion protection for practical applications.

As shown in Fig. 4b, with the increase in V_PEO_, the amplitudes of the potential oscillations decrease. The oscillations of potential exhibited the similar trends with the oscillations of the current, due to the initiation of localized corrosion and re-passivation. The more notable potential fluctuation for PEO-300 sample is mainly caused by the larger pore size of the PEO-300 coating compared to other coatings. Larger pore size leads to wider channels for electrolyte penetration and re-passivation, thus more significant change in the potential when such events occur. For the PEO-500 sample, even though the amplitude of the potential oscillations was smaller than that of the coated Al at lower voltages, the oscillations were still observed in the first 24 min. The initial corrosion potential was − 0.2 V, and decreased to approximately − 0.55 V afterwards (stabilized voltage data also shown in Table 1). When the coating voltage was increased to 600 V, the oscillation amplitude and time were largely reduced and shortened until the galvanic potential was stabilized to be about − 0.42 V. No oscillation can be observed in the case of PEO-700 (except the electrical noise) and the stabilized potential was − 0.36 V.

**Figure 5:**
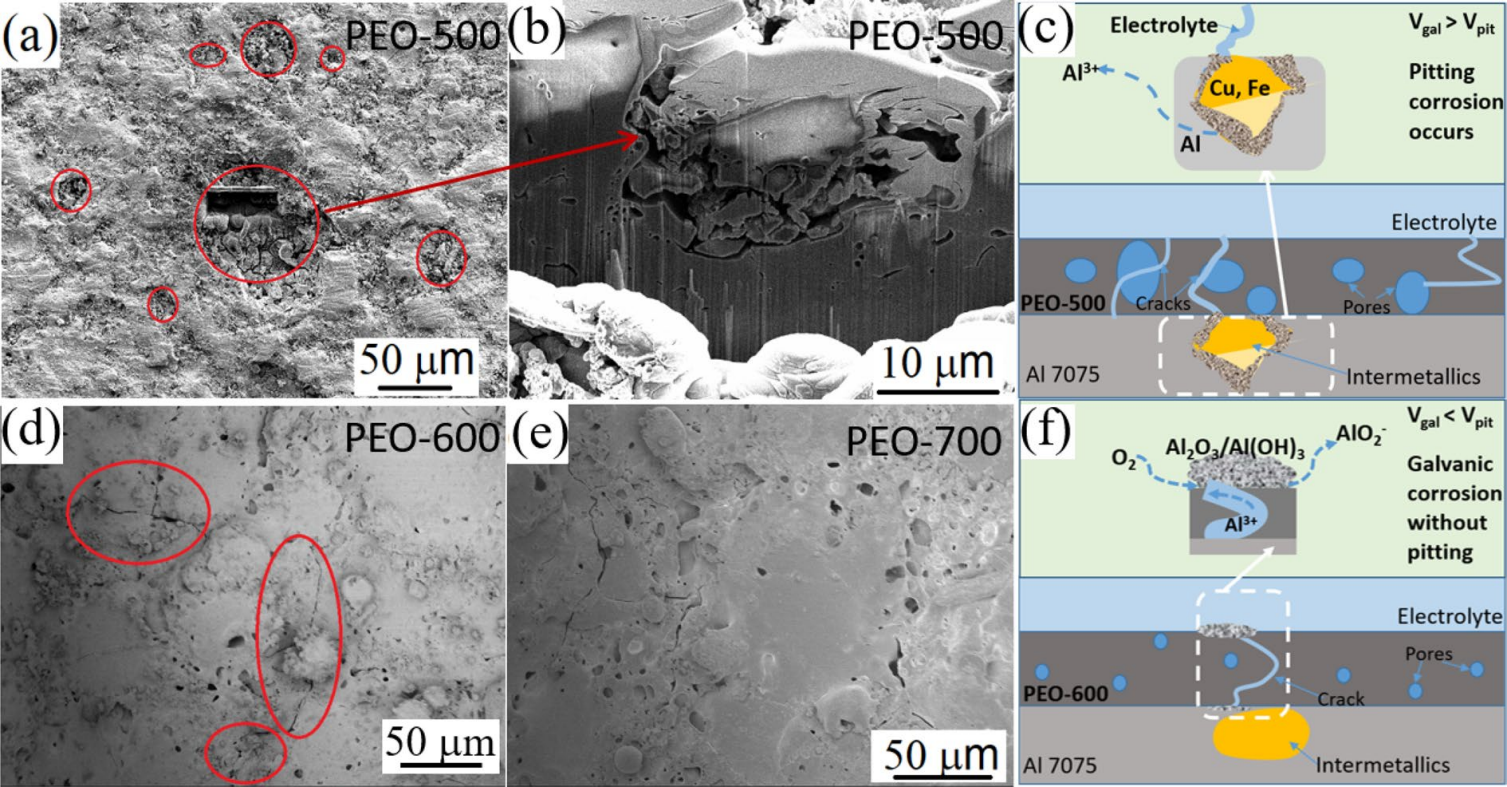
SEM images taken from (**a**) the surface and (**b**) cross section of FIB-cut corroded area of the PEO-500; The surface of (**d**) PEO-600 and (**e**) PEO-700 samples after galvanic coupling with CFRP in 3.5 wt% NaCl solution; Schematic diagrams showing the GC mechanisms of (**c**) PEO-500 and (**f**) PEO-600 samples;

Figure 5a shows the surface of PEO-500 after the galvanic coupling for two hours. Corrosion pits were clearly seen on the coating surface, and typical examples were highlighted in Fig. 5a by the red circles. Figure 5b shows a cross sectional SEM image taken from the specimen prepared by focused ion beam (FIB) at a typical corroded pit. As we discussed in the microstructure part (Fig. 3), the PEO-500 coating showed amorphous structure with some pores and cracks, which may provide some tunnels for the electrolytes thus initiating the pitting corrosion (Fig. 5c). However, such localized pitting disappeared for the PEO-600 and PEO-700 samples (Fig. 5d,e). Besides the reason of much more compact crystalized coating structure, this could also be explained by the difference in galvanic potentials. The pitting potential of Al 7075 in 3.5 wt% NaCl solution is − 0.55 V^38^, which is more negative than the stabilized galvanic potentials (Table 1) of PEO-600 and PEO-700 samples, i.e., − 0.42 and − 0.36 V, respectively. However, the morphology of the oxidation products on the surface of PEO-600 (circle highlights in Fig. 5d) indicates that besides the pitting damage, GC damage may also occur during the galvanic coupling with CFRP. As illustrated in Fig. 5f, the electrolyte may flow through a few micro-pores and micro-cracks forming the galvanic connecting between Al alloy and CFRP. Such connection may cause the crevice corrosion without pitting since the galvanic potential was lower than the pitting potential of Al 7075. During this process, the Al^3+^ ions transported through the cracks and oxidized on the coating surface. Compared with the PEO-600 sample, the PEO-700 sample exhibited much less oxidation products around the cracks and pores (Fig. 5e). It may be due to the much more compact crystalized structures of the ceramic coating. The reduced galvanic potentials, together with the surface characterizations, show that the PEO-700 coatings effectively hindered the pitting formation on the Al alloy and provide excellent protection during the Al-alloy/CFRP coupling.

**Figure 6:**
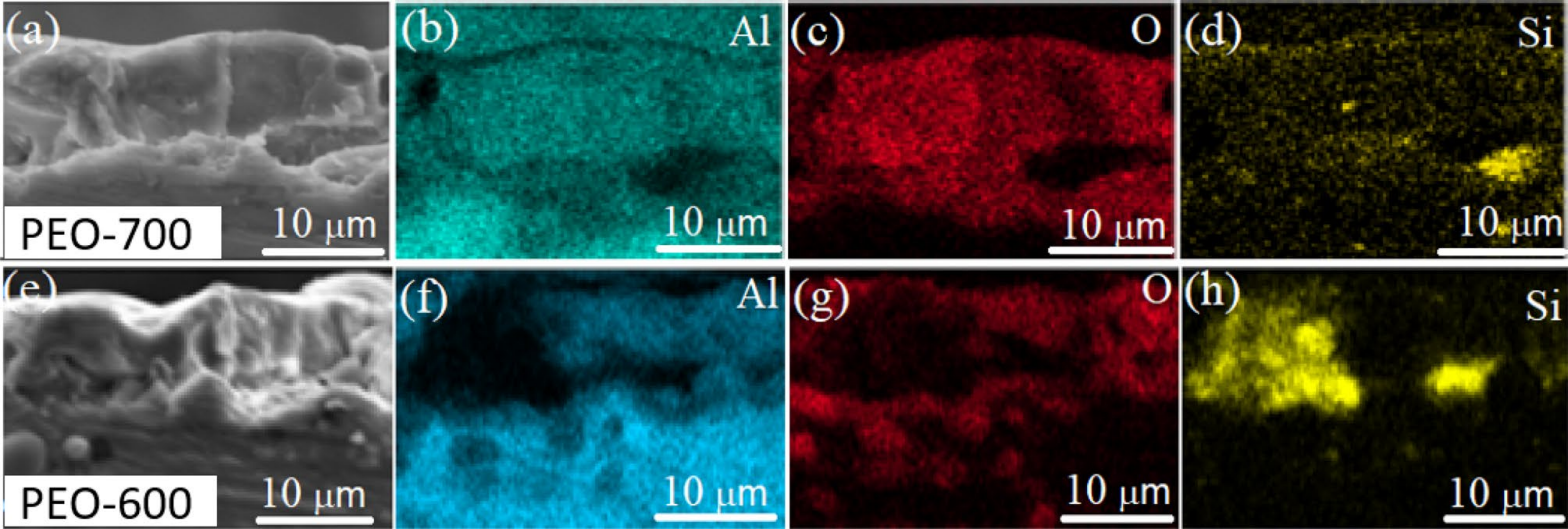
Cross section SEM images and EDX mappings of Al, O and Si recorded from the (**a**–**d**) PEO-700 sample and (**e**–**h**) PEO-600 sample.

The protection effect of PEO coating against GC can be explained in two aspects, one is the high insulation performance and the other is the anti-permeability^55^. The higher V_PEO_ contributes enhanced plasma and higher temperature during the PEO process that provides sufficient energy for phase transformations, resulting in more crystallized compact ceramic phase with improved insulation property (e.g., see the mullite in Fig. 3f). In addition, the super high temperature tends to accelerate the diffusions, speed up the reaction, and promote the melting of the surface oxides that, in turn, contributes to the uniformity of the element distributions and the formation of the compact structures. Figure 6 shows cross-sectional SEM images and EDX mappings taken from the PEO-600 and PEO-700 samples. The PEO-produced coating layer was about 10–11 μm thick for both samples. However, the Si distribution was much more uniform in PEO-700 than that in PEO-600. The uniform element distributions and the compact structures produced by the PEO process with increased V_PEO_ are believed to inhibit the GC.

## Conclusion

PEO process has been carried out on Al alloy at various voltages for its GC protections in contact with CFRP. Structural and electrochemical characterizations show that the PEO-600 and PEO-700 coatings are dominated by crystallized Al_2_O_3_ and mullite (i.e., 3Al_2_O_3_·2SiO_2_ and/or 2Al_2_O_3_·SiO_2_) phases. Due to the electrical insulation of these ceramic phases, the galvanic coupling between the CFRP and the PEO coated Al alloy base material tends to be isolated, which provides the corrosion protection. A comparison between the anodized coatings processed by the commercial TFSAA and the PEO methods reveals that the PEO process can significantly reduce the GC current density and the reductions can be up to ~ 90% by increasing the PEO voltage. However, microscale pores and cracks appeared in PEO-produced ceramic coatings with V_PEO_ ≤ 600 V, and they could decrease the corrosion protection effect of the coating. A high PEO process voltage of 700 V provides sufficient energy to produce uniform and crystalline structures that give rise to improved corrosion protections.

## Materials and methods

### Materials

The Al 7075-T6 alloy coupons of 2 mm thick were used in this study. The chemical compositions of the alloy are summarized in Table 2. The CFRP composite sheets, consisted of unidirectional carbon fibres (55 vol%) and epoxy, are also 2 mm in thickness. The interlacing structure of CFRP is shown in Fig. S3 of Supplementary Material. The surface of the Al 7075 T6 coupons was polished using SiC papers in the sequence of 400-, 600-, 800-, 1200-, and 2000-grit. After grinding, they were cleaned by ethanol and deionised water in sequence, followed by flowing air drying.

**Table 2:** Chemical composition of the Al alloy 7075-T6.

Element	Al	Zn	Mg	Cu	Fe	Mn	Cr	Others
Wt%	88.52%	6%	2.5%	1.6%	0.5%	0.3%	0.23%	0.37%

### Anodizing and PEO process

The Al anodization was carried out in a two-electrode cell system using a 15 vol% H_2_SO_4_ solution as the electrolyte and a Pt wire as counter electrode^56,57^. The anodizing voltage was set at 15 V for 20 min with the increment of 3 V/min. The PEO process was carried out using a specially designed AC (alternating current) high voltage power supply (manufactured by SOYIPOWER, China). The Al alloy coupon was used as the working electrode and a stainless steel 316 panel (40 mm × 20 mm × 3 mm) as the counter electrode. The distance between the two electrodes was fixed at 30 mm. The processing time was 20 min at an AC frequency of 50 Hz with the duty ratio of 30%. To study the effect of the applied voltage, PEO was processed at V_PEO_ = 300, 500, 600, and 700 V for the individual Al alloy coupon, and the coated samples were labelled as PEO-300, PEO-500, PEO-600, PEO-700, respectively. The electrolyte of the PEO process contains a mixture of 2 g/L KOH and 10 g/L Na_2_SiO_3_, the total volume is 1L. The temperature of the electrolyte was maintained at 33–35 °C throughout the PEO process using a heat exchanger. After the PEO process, the Al alloy coupons were cleaned using deionized water followed by air-blow drying.

### Electrochemical testing

The flow of the galvanic coupling current between the Al 7075 T6 and CFRP specimens was monitored using Metrohm Autolab (PGSTAT302N) with ZRA model. Ag/AgCl was used as the reference electrode. The current densities and relative potentials were collected at an interval of 0.5 s. The Al alloy coupon to be tested was sealed in a sample holder that has an exposed area of 1.0 cm^2^. The exposed area of the CFRP sheets in this test is 0.8 cm^2^. The distance between the Al alloy and the CFRP electrodes is 1.0 cm. All measurements were carried out in a 3.5 wt% sodium chloride solution (pH 6.5, 25 °C) that was kept in a container open to air and without stirring.

### Physical and chemical characterization

X-ray diffraction (XRD) characterizations were performed on Bruker D8 Advance with Cu-Kα X-ray source. While a JEOL Nova Nanosem 230 microscope was used for the scanning-electron microscopy (SEM) analysis and the energy dispersive X-ray (EDX) spectral analysis. Cross-section analysis was performed using a Helios NanoLab 450S dual beam focused ion beam instrument.

## Supplementary Information

Supplementary Information.
