# Supplementary material for: Galvanic corrosion protection of Al-alloy in contact with carbon fibre reinforced polymer through plasma electrolytic oxidation treatment
Source: Sci Rep. 2022 Mar 16;12:4532. doi: 10.1038/s41598-022-08727-7 (PMC8927304; doi:10.1038/s41598-022-08727-7)
Supplement: Supplementary file 1 — Supplementary Information. [file 41598_2022_8727_MOESM1_ESM.docx]

**Appendix A**

Supplementary Material

**Galvanic corrosion protection of Al-alloy in contact with carbon fibre reinforced polymer through plasma electrolytic oxidation treatment**

Junyi Liu, Xiaohu Huang, Yi Ren, Lai Mun Wong, Hongfei Liu, Shijie Wang^*^

*Institute of Materials Research and Engineering (IMRE), A*STAR (Agency for Science, Technology and Research), 2 Fusionopolis Way, Singapore 138634, Singapore*

* Corresponding author

Email: [*sj-wang@imre.a-star.edu.sg*](mailto:sj-wang@imre.a-star.edu.sg)


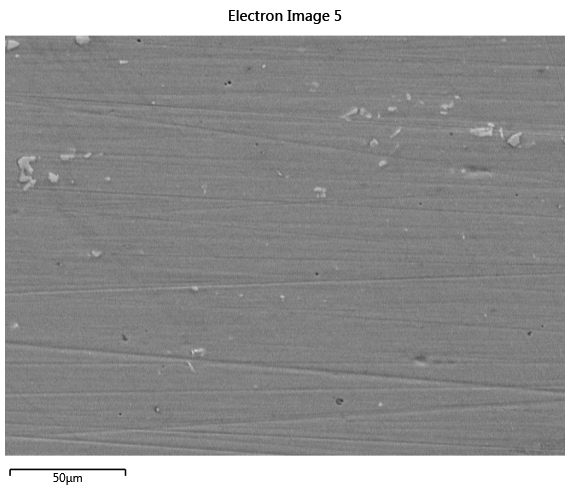

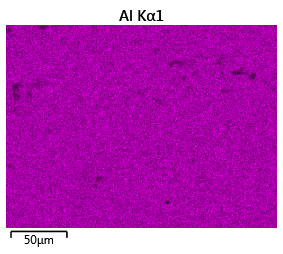

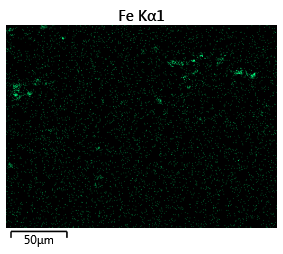


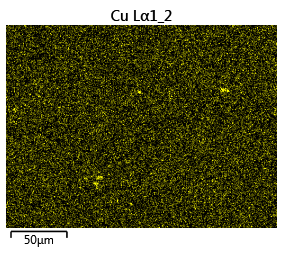

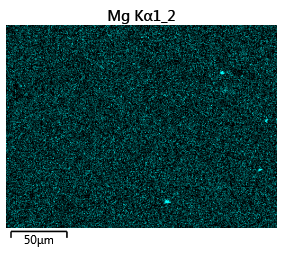


Fig S1. SEM images and EDX element mapping showing the distributions of intementallics highlighted in circles.


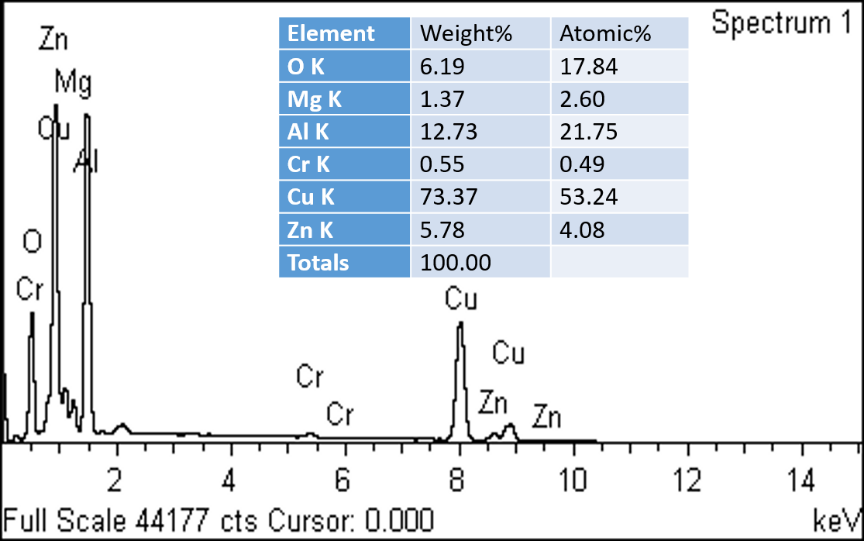


Fig S2. Element compositions from the EDX analysis of Spectrum 1.


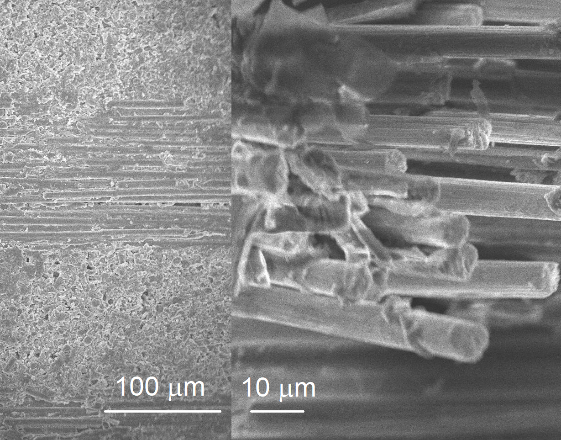


Fig S3. Microstructures of CFRP used in this work
